# High-speed metamagnetic switching of FeRh through Joule heating

**DOI:** 10.1038/s41598-022-26587-z

**Published:** 2022-12-21

**Authors:** Nicholas A. Blumenschein, Gregory M. Stephen, Cory D. Cress, Samuel W. LaGasse, Aubrey T. Hanbicki, Steven P. Bennett, Adam L. Friedman

**Affiliations:** 1 Laboratory for Physical Sciences, 8050 Greenmead Dr., College Park, MD 20740 USA; 2 Electronics Science and Technology Division, United States Naval Research Laboratory, 4555 Overlook Ave., SW, Washington, DC 20375 USA; 3 Materials Science and Technology Division United States Naval Research Laboratory, 4555 Overlook Ave., SW, Washington, DC 20375 USA

**Keywords:** Applied physics, Electronics, photonics and device physics, Electrical and electronic engineering

## Abstract

Due to its proximity to room temperature and demonstrated high degree of temperature tunability, FeRh’s metamagnetic ordering transition is attractive for novel high-performance computing devices seeking to use magnetism as the state variable. We demonstrate electrical control of the antiferromagnetic-to-ferromagnetic transition via Joule heating in FeRh wires. The magnetic transition of FeRh is accompanied by a change in resistivity, which can be probed electrically and allows for integration into switching devices. Finite element simulations based on abrupt state transition within each domain result in a globally smooth transition that agrees with the experimental findings and provides insight into the thermodynamics involved. We measure a 150 K decrease in transition temperature with currents up to 60 mA, limited only by the dimensions of the device. The sizeable shift in transition temperature scales with current density and wire length, suggesting the absolute resistance and heat dissipation of the substrate are also important. The FeRh phase change is evaluated by pulsed I-V using a variety of bias conditions. We demonstrate high speed (~ ns) memristor-like behavior and report device performance parameters such as switching speed and power consumption that compare favorably with state-of-the-art phase change memristive technologies.

## Introduction

Magnetic materials are crucial components of memory devices because of their inherent non-volatility, radiation hardness, and ease of control^1–4^. Using magnetization as a state variable has several advantages over charge-based devices^5^. For one, the resonant frequencies within magnetic materials are at least an order of magnitude faster than existing DRAM technology (*f* ~ 6400 megahertz for DDR5): gigahertz for ferromagnets, and terahertz for antiferromagnets^6,7^. Presently, commercially-available MRAM technology relies on spin-transfer torque to manipulate the magnetic moment direction and switch the free magnetic layer in magnetic tunnel junctions^8^. This requires a high current amplitude that can degrade the tunnel barrier rather quickly, rendering the device inoperable^9,10^.

An alternative approach to employ magnetization-based devices is to toggle the magnetization state itself by switching between a ferromagnetic (FM) and antiferromagnetic (AFM) phase, giving a clear on/off state in a phase-change device. Phase-change memory (PCM) devices are typically operated based on the resistivity contrast of insulating amorphous and conductive crystalline phases^11,12^. Indeed, PCM devices capable of faster write times and higher endurance than traditional NAND memory are within reach^12^. FeRh provides an ideal platform for fast, lithographically simple phase-change memories because of its AFM to FM transition that is accompanied by volumetric expansion of the CsCl-type crystal lattice and a significant change in resistivity^13–15^. FeRh switching is unique because it is based on a magnetic phase change, but functionally this manifests as a change in resistivity.

Furthermore, the critical temperature (*T*_*Cr*_), which we define as the start of the AFM-FM transition when heating, occurs is near room temperature and can be tuned using substitutional doping^16^, strain^7,17–27^, and patterning^24,28,29^. Because the transition is temperature-dependent, device manipulation through Joule heating is also possible^30^. An electric current flowing through an FeRh wire can heat the material above T_Cr_, inducing the AFM-FM transition^29,31–33^. Previous reports on FeRh suggest that the transition is very rapid, occurring on a time scale of ≤ 500 fs^34^. This could lead to a new class of PCM devices operating at THz frequencies perfectly suited to neuromorphic computing applications^35^.

In this work we demonstrate fast resistive switching of FeRh wires through Joule heating. We find the shift in AFM-FM transition temperature scales with both current density and wire geometry. We use pulsed I-V measurements to investigate the dynamic Joule heating effects and resulting power switching losses accompanying the AFM-FM transition. We obtain device switching speeds of about 300 ns, a value limited by our measurement equipment. We perform finite element method-based simulations to confirm the explanation for the observed behavior and provide further insight into the heat-induced transition.

## Methods

**Figure 1:**
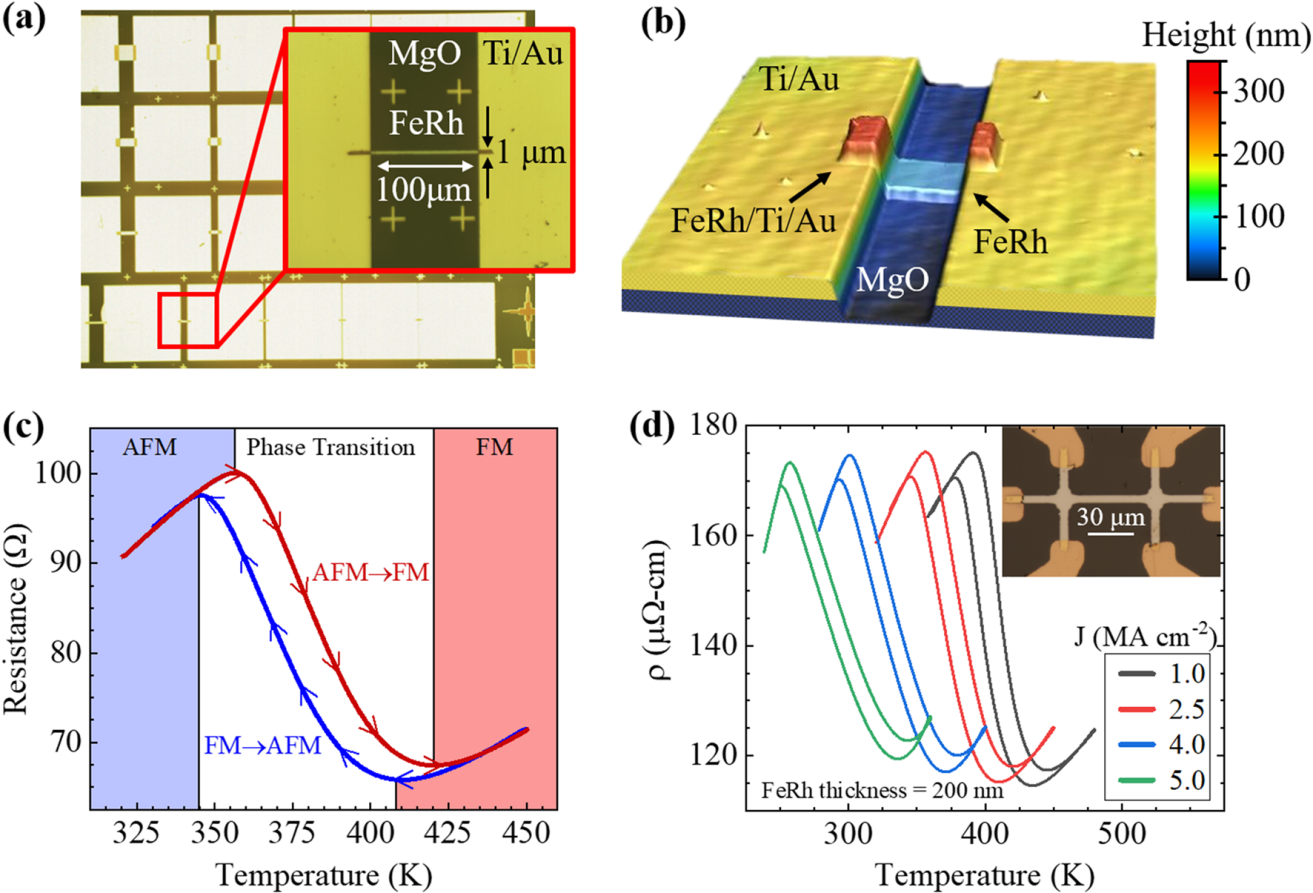
(**a**) Top-view optical images of the fabricated two-terminal FeRh devices with 35-nm-thick wires with widths and lengths varying from 0.3 to 50 µm and 2.5 to 100 µm, respectively. The enhanced image shows an FeRh wire with width, height, and thickness of 1 µm, 100 µm, and 35 nm, respectively. (**b**) Three-dimensional optical image of the device showing topology. (**c**) Proof-of-concept FeRh wire resistance while varying the ambient temperature from 320 to 450 K. Red and blue curves represent heating and cooling cycles, respectively. Background shading colors denote temperature regimes at which the FeRh is AFM (blue), FM (red), and in transition (white). (**d**) FeRh Hall bar resistivity as a function of ambient temperature while varying the current density through the device. The inset shows an image of the FeRh Hall bar with FeRh layer thickness of 200 nm.

FeRh films were grown on MgO substrates by sputter deposition with thicknesses of 35 and 200 nm. A 500 nm SiO_2_ layer was deposited using plasma-enhanced chemical vapor deposition (PECVD) as a hard mask for FeRh ion milling. Standard photolithography was used to define 10 × 100 μm Hall bars and wires of varying widths and lengths. Electron beam lithography (EBL) was used to define the wire geometry in poly-methyl methacrylate (PMMA) followed by metal deposition of a 20 nm Cr hard mask on the SiO_2_ surface. The SiO_2_ was then etched by inductively coupled plasma (ICP) reactive ion etching (RIE) with 10 sccm CHF_3_ and 15 sccm Ar with a chamber pressure of 30 mTorr, 30 W RF power, and 600 W ICP power, defining a hard mask for the ion milling process. The Cr mask was removed by the ion milling process and any remaining SiO_2_ over the FeRh wires is removed by a second ICP/RIE step. Finally, EBL was used to define bond-pads in PMMA followed by electron beam evaporation of Ti/Au (10/50 nm) and metal lift-off in acetone. Fabricated FeRh wires are shown in Fig. 1a,b.

The FeRh crystalline quality was evaluated using X-ray diffraction and high-resolution transmission electron microscopy, and the details for representative films have been previously reported elsewhere^36^. Resistance measurements were taken in a closed-cycle cryogenic probe station using a parameter analyzer. All measurement data was gathered while the sample was under a vacuum of < 1 × 10^–4^ Torr. COMSOL Multiphysics® software was used for finite element method simulation and analysis of the electro-thermal transport properties of FeRh and surrounding materials^37^.

## Results

FeRh films were processed into Hall bars and two-terminal wire devices with varying dimensions. Representative two-terminal devices are shown in Fig. 1a, with the enhanced image containing an FeRh device with a wire thickness, width, and length of 35 nm, 1 μm, and 100 μm, respectively. Figure 1b shows a three-dimensional optical image of the two-terminal FeRh device as measured using a confocal laser microscope. Figure 1c shows a behavioral schematic of an FeRh Hall bar with film thickness of 200 nm. Here, the resistance is plotted as a function of ambient temperature while applying a constant 25 mA current (2.5 × 10^6^ A cm^−2^). The background shading colors denote the three different temperature regimes at which the FeRh is AFM (blue), FM (red), and in transition (white). Upon heating the device (red curve), we see the FeRh begin the AFM-FM transition once the temperature exceeds T_Cr_ (T > 355 K). The phase transition is accompanied by a decreasing resistance, persisting until T > 420 K where the FeRh has fully transitioned into the FM phase. A similar-but-opposite effect occurs when cooling the device (blue curve; FM-AFM transition for 345 K < T < 410 K, AFM transition when T < 345 K). In Fig. 1d we display the resistivity (ρ_xx_) of a 200-nm-thick FeRh Hall bar (inset) as a function of temperature for current densities ranging from J = 1 × 10^6^ A cm^−2^ to 5 × 10^6^ A cm^−2^ (10 mA to 50 mA, respectively), measured while sweeping the sample temperature at a rate of ± 1 K min^−1^. The AFM-FM transition, as evidenced by the abrupt change in resistivity, is observed for each applied current density regardless of ambient temperature, but shifts from 410 K at 1 × 10^6^ A cm^−2^ to 255 K at 5 × 10^6^ A cm^−2^. The decrease in transition temperature is accompanied by a widening of the transition range that can be explained by our model, as discussed below and in the supplemental file S1.

**Figure 2:**
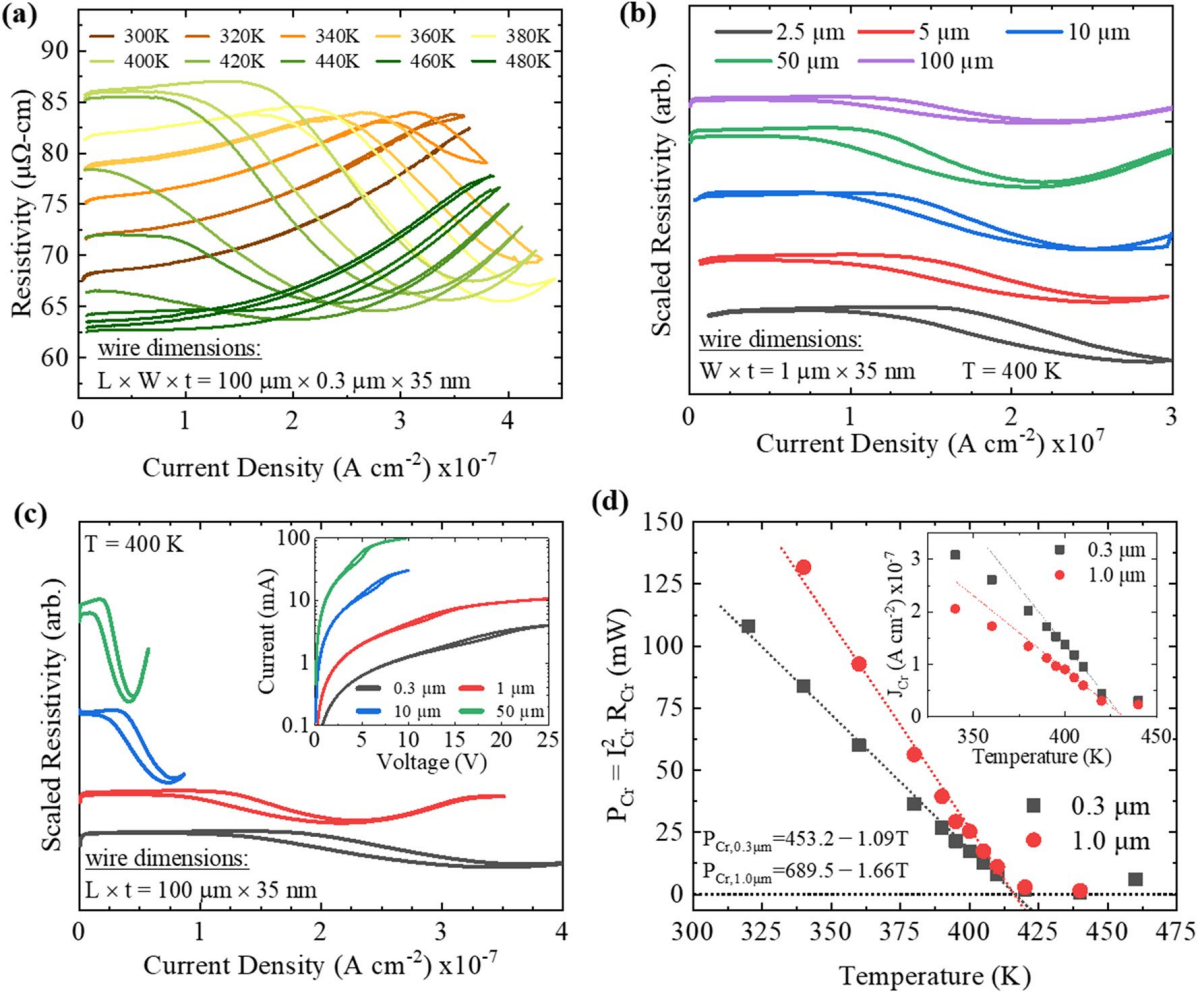
FeRh magnetic phase transition analysis by DC transport characterization to investigate temperature and geometrical dependencies. (**a**) FeRh resistivity as a function of current density for ambient temperatures ranging from 300 to 480 K. The experimental data shown here were obtained by measuring a FeRh wire with width, length, and thickness of 0.3 µm, 100 µm, and 35 nm, respectively. The critical current density, J_Cr_, decreased significantly as the ambient temperature was increased. Resistivity was then measured as a function of current density for a series of wires with varying (**b**) lengths and (**c**) widths. The wire-length-dependent ρ-J measurements were performed using a 1-µm-wide wire, while the wire-width-dependent ρ-J measurements were performed using a 100-µm-long wire. The inset of (**c**) shows the measured I-V characteristic for the wires of varying width. (**d**) Critical power dissipation is shown as a function of ambient temperature for 100-µm-long wires with widths of 0.3 µm (red squares) and 1 µm (blue circles). The inset plot in (**d**) shows the critical current density for the same two wires. Joule heating phenomenon was confirmed by fitting the P_Cr_-T data, during which an identical T_Cr_ of 415 K was extracted from the two wires with different geometries.

With the current-dependence of the AFM-FM transition established, we then investigated the transition at fixed temperatures while sweeping current in the FeRh wires. Figure 2a shows the wire resistivity as a function of current density at sample temperatures ranging from 300 to 480 K for a 0.3 × 100 µm wire with thickness of 35 nm. At 300 K the FeRh remains in the AFM phase as the induced Joule heating is insufficient to elevate the wire temperature to T_Cr_. At 320 K the AFM-FM transition begins at a high current density of ~ 3.3 × 10^7^ A cm^−2^. At 340–360 K we observe a larger region of the AFM-FM transition, but the current density is insufficient to force the FM transition. At 380–400 K the wire fully transitions into the FM phase, then when the current density is decreased the FM-AFM transition begins as the wire is cooled. At 420–440 K the wire begins in the AFM phase, then transitions into the FM phase with an increasing current density. However, the high ambient temperature prevents the FeRh from transitioning back into the AFM phase when reducing the current density to zero. At 460–480 K the wire begins in the FM phase, as the AFM-FM transition was fully driven by ambient temperature. The shift in transition temperature, measured as the maximum of the ρ-J curve where ρ begins to decrease, is shown in the inset of Fig. 2d. Joule heating depends on applied power and is directly proportional to the square of the current. Therefore, the reduced current density required for the AFM-FM phase transition at higher temperatures is a clear indication of Joule heating.

Wire geometry dependencies were investigated to further evaluate thermal dissipation effects. These measurements were performed at 400 K. Figure 2b shows the ρ-J curves for 1 µm wide FeRh wires with lengths ranging from 2.5 µm to 100 µm and thickness of 35 nm. The resistivity scale for each curve is offset for clarity. The current density through a wire does not scale as a function of wire length. Nonetheless, here we observe an AFM-FM transition temperature that is reduced for longer wires. Applying a constant current density through wires of increasing length requires a larger applied bias. Regardless of the associated thermal profile, longer wires dissipate more power which causes the substrate temperature to increase. Similarly, as shown in the ρ-J curves of Fig. 2c, the wire width also effects AFM-FM transition temperature. The data shown here were measured using 100 µm long FeRh wires with widths ranging from 0.3 to 50 µm. The curves are offset for clarity. The larger wires cannot cool as quickly, as a larger FeRh/substrate interfacial area results in a greater substrate heating contribution and lessened heat dissipation into the metal contacts and surrounding ambient. The 100 µm length was chosen because many of the shorter wires do not fully transition through the hysteresis region within the given current density range.

Figure 2d shows the critical power dissipation, *P*_*Cr*_, and critical current density, *J*_*Cr*_ (inset), as a function of temperature for two 100 μm long FeRh wires with widths of 0.3 μm (red squares) and 1.0 μm (blue circles). As shown in the inset, *J*_*Cr*_ does not decrease linearly with increasing temperature, consistent with a previous report^32^. However, a linear dependence is observed when plotting *P*_*Cr*_ vs. temperature. The critical power dissipation is calculated by

1 $$ P_{Cr} = I_{Cr}^{2} R_{Cr} \triangleq \alpha \Delta T, $$

which is expressed in terms of critical current (*I*_*Cr*_) and critical resistance (*R*_*Cr*_) at which the FeRh AFM-FM transition begins. Once the FeRh wire has reached thermal equilibrium, we define *P*_*Cr*_ = αΔT, where α [W K^−1^] describes heat dissipation from the FeRh wire and ΔT is the temperature increase caused by Joule heating. The linear dependence of *P*_*Cr*_ on *T* indicates that the AFM-FM phase transition is directly caused by Joule heating. Notably, an identical *T*_*Cr*_ of 415 K is observed for both wire dimensions when extrapolating *P*_*Cr*_ to the x-axis. The slope of the two *P*_*Cr*_ curves vary because of α, which is found to be − 1.09 W K^−1^ and − 1.66 W K^−1^ for wire widths of 0.3 μm and 1 μm, respectively. The observed increase of α amplitude for the larger wire width is a function of the FeRh/metal interfacial area, consistent with previous reports^32^.

Above and below the metamagnetic transition, the electrical transport properties follow that of typical metals, where increased phonon scattering degrades conductance leading to a positive temperature coefficient of resistance^38^. Moreover, the magnitude of the temperature coefficient of resistance is approximately equal in both the AFM and FM states. The absolute value of the resistivity, not its temperature dependence, differs. Near the metamagnetic transition temperature, microscopic domains of FeRh in the AFM state begin to transition into the FM state, simultaneously changing the overall resistance of the conductor. The precise temperature at which each domain changes is driven by defects, strain, the state of neighboring domains, etc., creating a distribution of temperatures over which the full transition occurs^39^. Therefore, for a single domain, the change in resistance with temperature would appear abrupt and square-like, while the distribution of transition temperatures in a multi-domain wire would induce a gradual change. The origin of these thermal mechanisms, as well as the observed Joule heating behavior, are elucidated via finite element simulations. Heat transfer physics were modeled using temperature-dependent thermodynamic (C_p_, κ, density) and electrical properties. In particular, the temperature-dependent conductivity of FeRh in the AFM and FM states was defined as shown in Fig. S1a. Additional modeling details are found in the supplemental file S1.

The simulated wire dimensions and biasing conditions (Fig. S1b) closely match those of Fig. 2a, and the simulation captures the primary experimental behavior, verifying the modeling approach. In particular, in the model we observe a hysteresis of approximately 10–15 K, a reduction in the onset of transition temperature with DC bias, and an increase in the transition width with DC bias, all of which are reflected in the measured results. In Fig. S1c,d we simulate the wire length and width dependencies that were measured experimentally and shown in Fig. 2b,c. These trends are consistent with the experimental data, where the ampacity significantly increases for the narrowest wire. This effect is caused by the ampacity scaling as a function of the wire linear mass density^36^.

**Figure 3:**
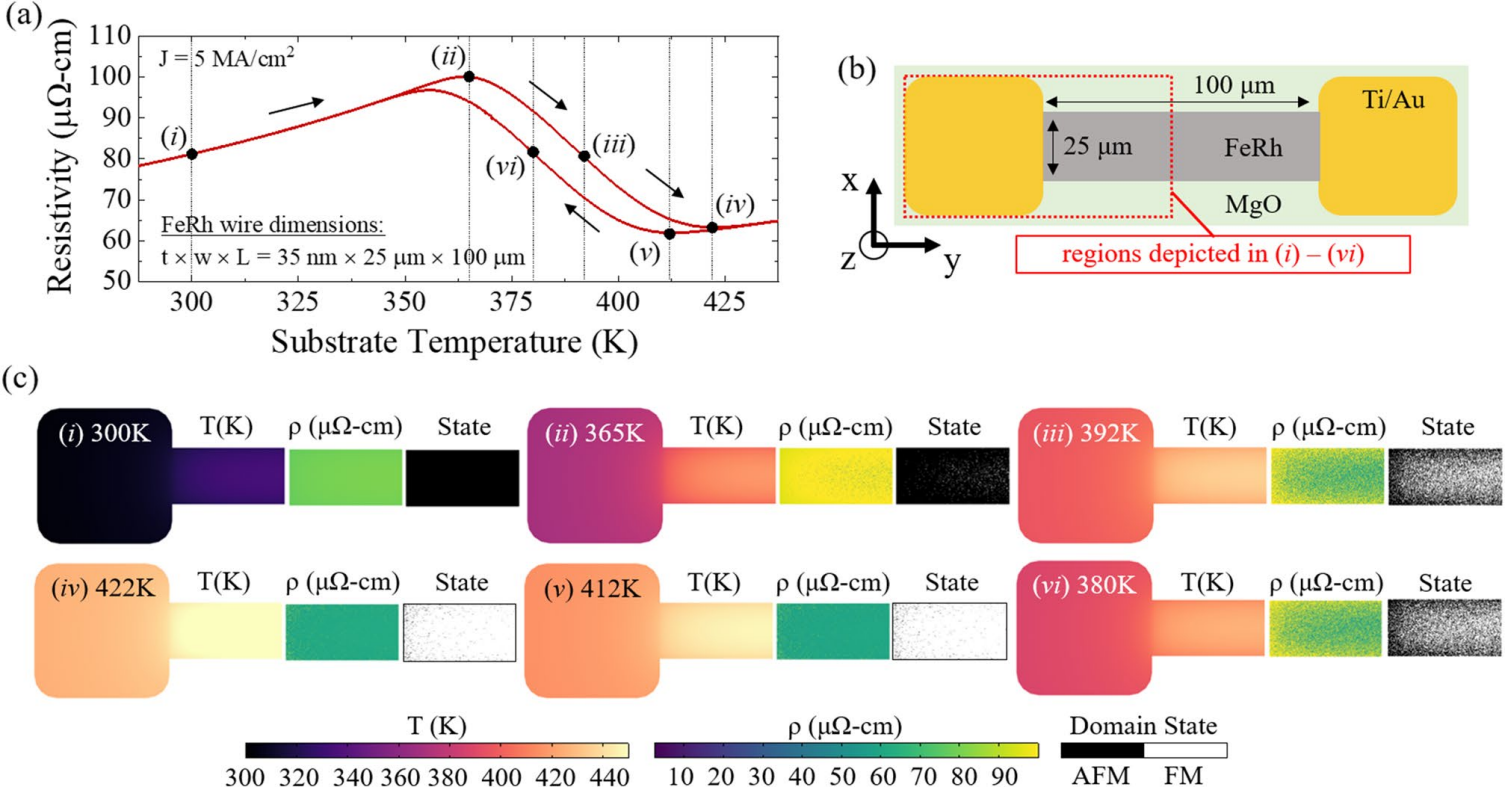
(**a**) Representative resistance vs. temperature sweep for a current density of 5 × 10^6^ A cm^−2^. Roman numeral labels indicate the specific temperatures at which the subsequent surface maps were generated. The surface maps were generated while sweeping the temperature during a heating cycle, as indicated by the data points shown on the curve. (**b**) A schematic diagram of the FeRh wire device including a region outlined with a red dotted line to indicate the portion of the sample which is depicted in the subsequent surface maps, including half of the FeRh wire length one metal contact. (**c**) Surface maps of the outlined sample area show temperature, wire resistivity, and individual FeRh domain states (AFM or FM) for substrate temperatures of (i) 300 K, (ii) 365 K, (iii) 392 K, (iv) 422 K, (v) 412 K, and (vi) 380 K. For (v) and (vi) the substrate is cooled from 500 K, all others are for the heating phase of the heat/cool cycle. The state of the domains (AFM/FM) and corresponding local resistivity correlate closely with the thermal profiles shown in the supplemental file (Fig. S2).

Analysis of the model parameters during a temperature sweep provides insight into the fundamental origins of the globally measured transport properties. Figure 3a represents wire resistivity as a function of substrate temperature for a current density of 5 × 10^6^ A cm^−2^. Figure 3b shows the device regions depicted in (i)–(vi), consisting of one metal contact and half of the 100-μm-long FeRh wire. In Fig. 3c we show surface maps of temperature, resistivity, and state of individually modeled FeRh domains for substrate temperatures of (i) 300 K, (ii) 365 K, (iii) 392 K, (iv) 422 K, (v) 412 K, and (vi) 380 K. In (i), despite a relatively low substrate temperature of 300 K, the wire temperature increases to 334 K near the center. The elevated temperature is an effect of Joule heating, and it persists until reaching the edge of the contact where heat dissipation is enhanced by the Au electrode. Once the substrate temperature is raised to 365 K (ii), the peak wire temperature and terminal resistivity reach 417 K and 100 μΩ-cm, respectively. This temperature corresponds with the peak wire resistance, where temperature-induced increases in resistance are offset by the accumulation of domains which have transitioned to the lower resistivity FM state. These transitioned regions are observed as green domains and white domains in the resistivity and state maps in (ii), and are most concentrated near the middle of the FeRh wire where the temperature is the highest. This behavior becomes more pronounced as the temperature is increased to 392 K (iii). The locations of the transitioned regions are random, yet generally located near the wire center and away from the metal contacts, which are cooler because of high thermal conductivity. At 422 K (iv), the wire reaches its minimum resistance state, where the temperature-induced increases in resistance exceed the reduction in resistance from switching domains, since nearly all domains are now in the FM state. The hysteresis in the wire is clearly evidenced on the cooling cycle as nearly all domains remain in the same state at 412 K (v), and despite having a 12 K cooler substrate at 380 K (vi) we observe resistivity and state maps that closely resemble those observed upon heating to 392 K (iii).

**Figure 4:**
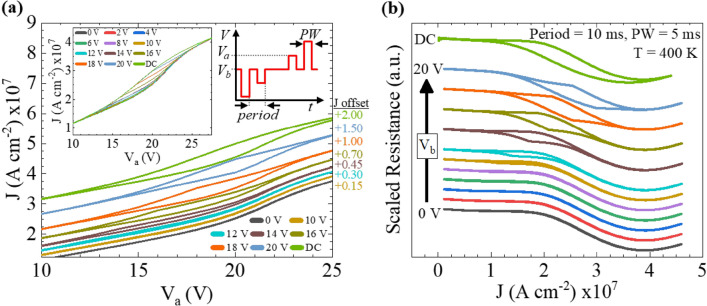
A FeRh wire was characterized by pulsed J-V to investigate operating conditions which are more coherent with switching devices used in practice. The data shown here were acquired at an ambient temperature of 400 K using a FeRh wire with length, width, and thickness of 100 μm, 0.3 μm, and 35 nm, respectively. (**a**) J-V_a_ and (**b**) R-J curves are shown while varying the base voltage amplitude. Aside from the inset plot of (**a**), each curve is offset for clarity and both plots contain a DC measurement for comparison purposes. Data were gathered using bias pulses of 10 ms period and 5 ms pulse width. The inset plot in (**a**) contains the as-measured J-V curves without an offset to show how they collapse onto a single loop. Additionally, a pulse profile schematic containing the pulse width (PW), baseline voltage (V_b_), and amplitude voltage (V_a_) is shown in (**a**).

A pulsed bias allows more precise control over self-heating effects and establishes realistic device operation parameters such as switching speeds. Pulsed I-V has been used to analyze thermal effects in a wide variety of materials, including Si^40^, wide bandgap semiconductors such as GaN and Ga_2_O_3_^41,42^, and phase change materials like Ge_3_Sb_2_Te_6_^43^. Pulsed J-V measurements shown in Fig. 4a were made at 400 K on a FeRh device with length and width of 100 μm and 0.3 μm, respectively. The pulse profile, shown schematically in Fig. 4a includes a 5 ms pulse width (*PW*), 10 ms period, a pulsed amplitude voltage (*V*_*a*_) varied from 0 to 30 V and a baseline voltage (*V*_*b*_) varied from 0 to 20 V. The J-V curves are offset for visual clarity when *V*_*b*_ = 0 V and 10–20 V. A DC measurement is included for comparison purposes. The inset presents the data without the offset to show how the data collapses onto a single J-V loop. The hysteresis regions of the J-V curves remain fixed along the voltage axis, but widen along the current axis as *V*_*b*_ is increased from 10 to 20 V.

Figure 4b shows the change in resistance with current density (R-J), offset for clarity. Hysteresis in the R-J characteristic begins to develop once *V*_*b*_ > 10 V. Although hysteresis is always seen in the DC measurements, the absence of hysteresis for low *V*_*b*_ during pulsed operation indicates that heat is dissipating at a high enough rate to allow the FeRh to cool and transition back into the AFM phase between each pulse. This thermal dissipation effect can be manipulated by increasing the baseline voltage, evident by the evolution of the R-J hysteresis with bias voltage. When using pulses, a persistent FeRh FM state is only achieved when the *V*_*b*_ amplitude is large enough such that the FeRh temperature exceeds *T*_*Cr*_ during standby operation. Otherwise, the FeRh temperature fall below *T*_*Cr*_ and the FeRh will transition into the AFM state.

**Figure 5:**
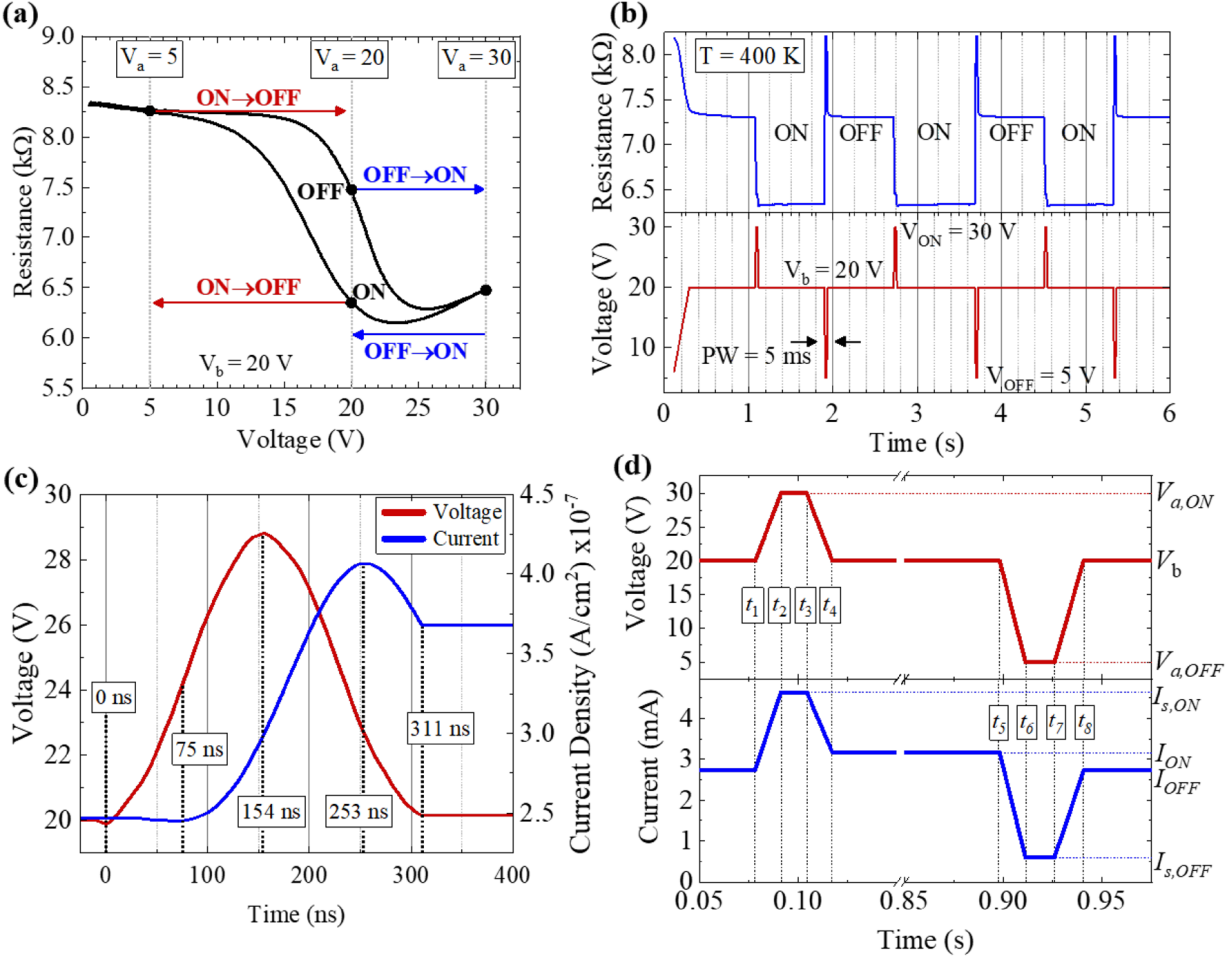
(**a**) Pulsed operation state-switching schematic diagram that shows the pulsed voltage amplitudes required to switch between AFM and FM states. At a constant 20 V bias, the FeRh can be in either of the two states, and it will remain in that state assuming it is thermally stabilized. Switching between states can be achieved by applying a short, pulsed voltage of either V_a_ = 5 V (e.g. FM-AFM transition, ON to OFF) or V_a_ = 30 V (e.g. AFM-FM transition, OFF to ON). In (**b**) we demonstrate this switching capability by showing the transient resistance of the FeRh while pulse biasing the device to switch between ON and OFF states using a V_b_ of 20 V and V_a_ of either 5 V (OFF) or 30 V (ON). (**c**) A high switching speed of 311 ns was observed when switching from OFF to ON. The switching speed shown here was limited by measurement equipment, and should serve as a ‘ceiling’ for FeRh switching speed capability. (**d**) Switching parameters used for calculating device power consumption, including *V*_*a,ON*_ = 30 V, *V*_*b*_ = 20 V, *V*_*a,OFF*_ = 5 V, *I*_*s,ON*_ = 4.646 mA, *I*_*ON*_ = 3.155 mA, *I*_*OFF*_ = 2.736 mA, *I*_*s,OFF*_ = 0.610 mA, *t*_*2*_–*t*_*1*_ = 13.045 ms, *t*_*3*_–*t*_*2*_ = 13.014 ms, *t*_*4*_–*t*_*3*_ = 12.999 ms, *t*_*6*_–*t*_*5*_ = 13.053 ms, *t*_*7*_–*t*_*6*_ = 14.753 ms, and *t*_*8*_–*t*_*7*_ = 14.742 ms.

The pulsed voltage conditions can be fine-tuned to quickly switch between the AFM and FM states. An example of this methodology is shown using the DC-IV plot of Fig. 5a for FeRh wire device (width = 0.3 µm, length = 100 µm). To function as a switch, the FeRh temperature must be stabilized such that the resistance lies somewhere within the metamagnetic transition region. For example, this could be achieved using a 20 V DC bias. Now, rather than applying a constant DC bias, we can apply a pulsed bias with a 20 V baseline voltage where the FeRh settles to a temperature within the metamagnetic transition region. At this temperature, crystal domains can exist in either an AFM (high resistance) or FM phase (low resistance). The system does not need to completely switch to a pure AFM or FM phase, it just needs to have a majority of domains in either the AFM or FM phase to produce a sufficient change in resistance. When *V*_*a*_ is set to 20 V, the AFM and FM phases have resistances of 7.4 kΩ and 6.3 kΩ, respectively. For example, the device can be switched ON (AFM-FM transition) by applying a pulsed voltage of *V*_*a*_ = 30 V then reducing *V*_*a*_ back to 20 V to maintain state (blue arrow). Likewise, it can be switched OFF (FM-AFM transition) by applying a pulsed voltage of *V*_*a*_ = 5 V then increasing *V*_*a*_ back to 20 V to maintain state (red arrow).

In Fig. 5b we demonstrate switching functionality using the methodology described above. The transient resistance (top blue curve) was measured while applying a pulsed voltage waveform (bottom red curve). The pulse profile consists of a 20 V baseline voltage, and short 5 ms voltage pulses to either 5 V or 30 V to switch between OFF and ON states, respectively. First, the voltage was ramped from 0 to 20 V to enter the hysteresis center-region and held at 20 V for ~ 1 s, maintaining the AFM state. At *t* = 1.1 s, the voltage is then pulsed to *V*_*a*_ = 30 V for 5 ms, causing the wire temperature to increase such that the AFM-FM transition occurs and the device is switched ON. After this 5 ms pulse, *V*_*a*_ is reduced to 20 V to maintain the FeRh temperature and hold the device in the ON state. Next, at *t* = 1.9 s, the device is switched OFF by applying a 5 ms pulse of *V*_*a*_ = 5 V, then returning to *V*_*a*_ = 20 V to maintain state. We find that ΔR/R_min_ = 16.4% when switched into the OFF state, which is ~ 3 × more resistance modulation than what has been reported for strain-based FeRh switching^7,26^. Device switching endurance and state retention were also evaluated and the results are shown in Fig. S4.

To establish an upper bound on the switching speed, we investigated the transient current measured during individual 150 ns voltage pulses (our instrumentation allows for single transient pulses for PW < 5 ms rather than a repeating waveform as shown above for PW > 5 ms). Figure 5c shows the applied transient voltage (red curve) and measured current (blue curve) during individual pulses where *V*_*a*_ and *V*_*b*_ were 29 V and 20 V, respectively. Within the resolution of our measurement, the device was capable of switching from OFF to ON in 311 ns. During this measurement, the equipment was configured to apply a 150 ns voltage pulse. However, the pulsed voltage had rise/fall times of ~ 150 ns, resulting in a pulse width of more than 300 ns, thus limiting our measurement capability. Therefore, this is an upper bound of the switching time, and we expect actual switching speeds to be much faster. Optical measurements by Pressacco, et al. showed the FeRh phase transition occurs on sub-picosecond time scales, suggesting that the operational limit for devices based on the FeRh transition could exceed GHz operating speeds, provided sufficient thermal sinking by the substrate^34^. To understand how this device can be optimized, we evaluate the power switching losses that occur during phase transition. Power consumption was calculated for the ON state and OFF states,

2 $${P}_{ON}={V}_{b}{I}_{ON},$$

3 $${P}_{OFF}={V}_{b}{I}_{OFF},$$

and also during turn-ON and turn-OFF,

4 $${P}_{s,ON}=\frac{1}{{T}_{s,ON}}\left[\left({t}_{2}-{t}_{1}\right)\left({I}_{s,ON}-{I}_{OFF}\right)\left({V}_{a,ON}-{V}_{b}\right)+\left({t}_{3}-{t}_{2}\right){I}_{s,ON}{V}_{a,ON}+\left({t}_{4}-{t}_{3}\right)\left({I}_{s,ON}-{I}_{ON}\right)\left({V}_{a,ON}-{V}_{b}\right)\right],$$

5 $${P}_{s,OFF}=\frac{1}{{T}_{s,OFF}}\left[\left({t}_{6}-{t}_{5}\right)\left({I}_{ON}-{I}_{s,OFF}\right)\left({V}_{b}-{V}_{a,OFF}\right)+\left({t}_{7}-{t}_{6}\right){I}_{s,OFF}{V}_{a,OFF}+\left({t}_{8}-{t}_{7}\right)\left({I}_{OFF}-{I}_{s,OFF}\right)\left({V}_{b}-{V}_{a,OFF}\right)\right]$$

switching cycles^44^. Here, *T*_*s*_ is the switching pulse period, *t*_*i*_ represents the pulsed waveform timings, *I*_*ON*_ and *I*_*OFF*_ are steady state current amplitudes, *I*_*s,ON*_ and *I*_*s,OFF*_ are switching current amplitudes resulting from pulsed voltages *V*_*a,ON*_ and *V*_*a,OFF*_, respectively. The value of each parameter is shown in Fig. 5d. Using these parameters, *P*_*ON*_, *P*_*OFF*_, *P*_*s,ON*_, and *P*_*s,OFF*_ were found to be 63.11 mW, 54.76 mW, 57.78 mW, and 23.82 mW, respectively.

## Discussion

Based on the switching properties observed in this work, FeRh is comparable to or better than many other candidate phase-change memory materials. Among these, Ge_2_Sb_2_Te_5_ is the most commonly used active layer material in phase change memory applications, and has reported read/write times of 150–200 ns^45,46^. TiO_x_ is commonly used for developing memristor networks requiring more complex architectures because it can provide low variability across the area of a chip^47^. Alibart, et al*.* fabricated a TiO_2_-based memristor that had an R_OFF_/R_ON_ and write time of 10 and 200 ns, respectively^48^. HfO_2_ is another commonly used memristor active layer material because of high R_OFF_/R_ON_ and turn-on slopes of over ten orders of magnitude and 1 mV/decade, respectively^49^. Despite the more intricate architectures of these memory devices, the 311 ns write time demonstrated in this work is comparable in switching speed performance.

Our rudimentary devices will require optimization to maximize R_OFF_/R_ON_, minimize the relatively high steady-state power consumption (*P*_*OFF*_ = 54.763 mW and *P*_*ON*_ = 63.105 mW), and ultimately achieve sub-ns switching speeds. There are several strategies for achieving these gains. The R_OFF_/R_ON_ ratio could be magnified by incorporation into tunneling magnetoresistance type sensors, where the dependence of the tunneling mechanism on the magnetic properties can result in orders of magnitude larger MR changes^50^. The power consumed is dominated by *V*_*b*_ since the pulsed waveform has such a small duty cycle, and the baseline voltage is necessary since it keeps the device temperature near the phase transition. By tuning the width and temperature onset of the FeRh transition, as well as the wire geometry, the required baseline voltage can be drastically reduced. This would also allow for a reduced ON-state power as a smaller pulse would be needed to switch states. The transition temperature of FeRh could also be tuned to lower temperatures by altering the Fe_x_Rh_1-x_ composition during growth. It was shown previously that the transition temperature can be controlled over a 80 K range by varying the Fe content from *x* = 0.40–0.49 or by He-ion implantation^25,51,52^. This would greatly reduce the required *V*_*b*_ and hence the static power consumption. As an example of a potential enhancement in device operating properties, in the current devices, the ON-state power could be reduced by ~ 32% to 43 mW by lowering *V*_*b*_ to 15 V.

The switching speeds of the devices reported here is comparable to that of conductive filament memristors, which can range from milliseconds to nanoseconds and varies depending on the thickness of the switching medium layer^53–55^. Further reducing the pulse width to the transition speed limit could result in probabilistic switching, allowing for applications of these devices in neuromorphic computing. Existing neuromorphic transistors operate with speeds from kilohertz up to megahertz^56–58^. The switching speed of the preliminary device presented here is therefore at least as fast as the state-of-the-art. The 400 K operating temperature used for device proof-of-concept in Fig. 5 was chosen to simulate a realistic operating condition in computing applications. However, in neuromorphic applications, the ideal operating temperature in an advanced system would likely be closer to 310 K^59^. This would have the added benefit of a lower power device. Further, the FeRh device presented here has similar functionality to Mott transistors, which operate based on thermally-induced phase change and can be switched on a sub-ns timescale^60–62^. Although our measurements were all done in vacuum, similar to other materials used for thermally-induced phase change devices, FeRh is unlikely to experience oxidation effects within the heating/cooling range implemented in this work (i.e. 400 K plus the Joule heating contribution). Because there are no stable compounds containing Fe, Rh, and O^63^, oxidation is only possible when the Fe-Rh bond is broken. The Fe-Rh bond has a formation energy of − 0.055 eV, which is equivalent to the energy provided by a temperature of 638 K. Since the experiments performed in this work were at temperatures less than or equal to 480 K, it is unlikely that any Fe-Rh decomposition would occur. Additionally, based on our findings, it would be possible to fabricate a nonvolatile FeRh device capable of storing memory at zero current which can only be erased by cooling.

## Conclusion

Our comprehensive results establish the feasibility of employing the metamagnetic transition in FeRh as the basis for a very fast, phase-change switch in future computing applications. Joule heating of FeRh wire devices was demonstrated and the geometrical dependencies on the metamagnetic AFM-FM phase transition were investigated. COMSOL® simulations and pulsed I-V measurements were used to evaluate the underlying thermal mechanics present during the AFM-FM transition. We demonstrated metamagnetic resistive switching capability with a switching speed of at least 311 ns. Future work on FeRh switching devices should address critical temperature tuning through growth such that *T*_*Cr*_ is near room temperature, as this would greatly increase device efficiency.

## Supplementary Information

Supplementary Information.

## Data Availability

The datasets generated during and/or analyzed during the current study are available from the corresponding author on reasonable request.
