# Supplementary material for: High-speed metamagnetic switching of FeRh through Joule heating
Source: Sci Rep. 2022 Dec 21;12:22061. doi: 10.1038/s41598-022-26587-z (PMC9772412; doi:10.1038/s41598-022-26587-z)
Supplement: Supplementary file 1 — Supplementary Information. [file 41598_2022_26587_MOESM1_ESM.docx]

**Supplementary Information**

**High-speed metamagnetic switching of FeRh through Joule heating**

Nicholas A. Blumenschein^1*^, Gregory M. Stephen^1^, Cory D. Cress^2^, Samuel W. LaGasse^2^, Aubrey T. Hanbicki^1^, Steven P. Bennett^3^, and Adam L. Friedman^1*^

^1^ Laboratory for Physical Sciences, 8050 Greenmead Dr., College Park, MD, 20740, USA

^2^ Electronics Science and Technology Division, United States Naval Research Laboratory, 4555 Overlook Ave., SW, Washington, DC, 20375, USA

^3^ Materials Science and Technology Division United States Naval Research Laboratory, 4555 Overlook Ave., SW, Washington, DC, 20375, USA

*^*^* nblumenschein@lps.umd.edu, afriedman@lps.umd.edu

**Thermal Model Description.**

Figure S1a shows the temperature-dependent conductivity functions used to model the heat transfer physics. Here, the temperature-dependent conductivity of FeRh in the AFM and FM states is defined as shown by the blue and green curves, respectively. These functions were obtained by fitting the temperature-dependent conductivity of FeRh in the respective states at temperatures above and below the metamagnetic transition temperature. To transition between the two states, a boundary ordinary differential equation constraint was defined in the model which set a state variable to 1 or 0 corresponding with the AFM and FM states, respectively. A value of 1 causes the domain to follow the AFM temperature-dependent conductivity function while a value of 0 causes it to follow the FM temperature-dependent conductivity function; transition between a state of 0 and 1 is abrupt and solely dependent on the transition temperature of the domain. The transition temperature of each domain was defined by three parameters. The first two parameters are global and define the transition from AFM to FM (430 K) along with the transition from FM to AFM (420 K). The difference between these two parameters gives rise to hysteresis in the metamagnetic transition temperature. Additionally, a random variable is assigned to each FeRh domain, that is seeded by the domain coordinates to keep it fixed for each sampling. Each domain is then sampled from a standard normal distribution, yielding a mean temperature shift of 0 K with ±1 standard deviation corresponding with a ±10 K shift. This random parameter causes each FeRh domain to transition at different temperatures, yielding a gradual change in the total wire resistance that is sampled at the wire terminals. Programmatically, FeRh wire and metal contacts were treated as layered materials, thus accounting for their thickness analytically thereby avoiding the need to mesh them in the out-of-plane direction. Therefore, each mesh element in the FeRh wire region was treated as an individual domain with the maximum domain size ranging from 100 nm to 250 nm.

In the model we chose distinct thermal conductivity and heat capacity parameters for each material. The FeRh thermal conductivity was recently investigated by Jiméneza et al. using first-principle calculations [63]. At 400 K the thermal conductivity varies from 31.8 W/m-K to 65.8 W/m-K for antiferromagnetic and ferromagnetic FeRh, respectively. In this work, we use a thermal conductivity value of 50 W/m-K since the FeRh device is mainly operated within the AFM-FM transition range. The FeRh heat capacity is set to vary with temperature according to

${Cp}_{FeRh}\left[ \frac{J}{kg K} \right]=252+6.3\left( T-150 \right)^{0.95}, (6)$

which was found by extrapolating data from a report by Cooke *et al.* [64], and agrees with additional findings published elsewhere [65]. The thermal conductivity,

$$\kappa_{MgO}\left[ \frac{W}{m K} \right]=\frac{14905\frac{W}{m}}{T}-\left( 0.10272\frac{W}{m} \right)T^{0.41267}, (7)$$

and heat capacity,

${Cp}_{MgO}\left[ \frac{J}{kg K} \right]=47.260+5.682\frac{T}{{10}^{3}}-0.873\left( \frac{T}{{10}^{3}} \right)^{2}+0.104\left( \frac{T}{{10}^{3}} \right)^{3}-1.054\left( \frac{{10}^{3}}{T} \right)^{2}, (8)$

of the MgO substrate varied with temperature according to previous findings [66]. The thermal conductivity and heat capacity of the gold contacts were 310 W/m-K and 128 J/kg-K, respectively [67].

Figure S1b shows the resistance of a 10 μm x 100 μm wire under different DC biases as the substrate temperature is increased and serves to verfiy the modeling approach. The simulated wire dimensions and biasing conditions closely match those of Fig. 2a, and indeed the simulation captures the primary behavior observed in experiment. In particular, we observe a hysteresis of approximately 10 – 15 K, a reduction in the onset of transition temperature with DC bias, and an increase in the transition width with DC bias, all of which are reflected in the measured results. The temperature-dependent behavior of the FeRh wire was also found to be highly dependent on the ability of the substrate to dissipate current-induced heating. The inset images in Fig. S1b show volume temperature contour plots within the MgO substrate for the FeRh wire while under a fixed current load of 4 MA/cm^2^ at different substrate temperatures as labeled. For the 1.0 MA/cm^2^ condition, the substrate temperature at which the minimum resistance occurs is approximately 440 K. This is approximately 2 standard deviations above the mean transition temperature and correlates to a FM domain transition percentage of >97%. At 2.5 MA/cm^2^, 4.0 MA/cm^2^, and 5.0 MA/cm^2^, the substrate temperature at which the minimum terminal resistance occurs decreases from 415 K to 375 K to 340 K, respectively. Simultaneously, the thermal profile originating at the FeRh wire and extending down into the substrate is clearly apparent. More rapid thermal equilibration within the substrate would lessen the thermal gradient experienced in the wire and concomitantly reduce the amplitude of Joule-heating-induced shift of the minimum resistance temperature. The opposite is also true, whereby a slower thermal equilibration would yield even greater thermal gradients at the expense of slower thermal constants for the wire/substrate system.

In Fig. S1c the simulated wire resistance is plotted as a function of current density for 1-μm-wide wires with lengths of 2.5 μm, 5 μm, 10 μm, and 50 µm. Consistent with the experimental data in Fig. 2b, the simulated wires show a reduction in the current at which the minimum conductivity is observed and a decreased *J_Cr_*, signifying the onset of the metamagnetic transition. At only 1 µm wide, the shorter wires also begin to display stepwise resistance fluctuations since the transition of individual domains provide greater contributions to the overall resistance. In Fig. S1c a maximum domain size of 100 nm was used to ensure the width of the simulated wire contained at least 10 elements. For Fig. S1b a maximum domain size of 250 nm was used since the wires were 10 µm long and a fixed length of 100 µm. Figure S1d shows the simulated wire resistance plotted as a function of current density for 100-µm-long wires with widths of 0.3 µm, 1.0 µm, 10 µm, 25 µm, and 50 µm.


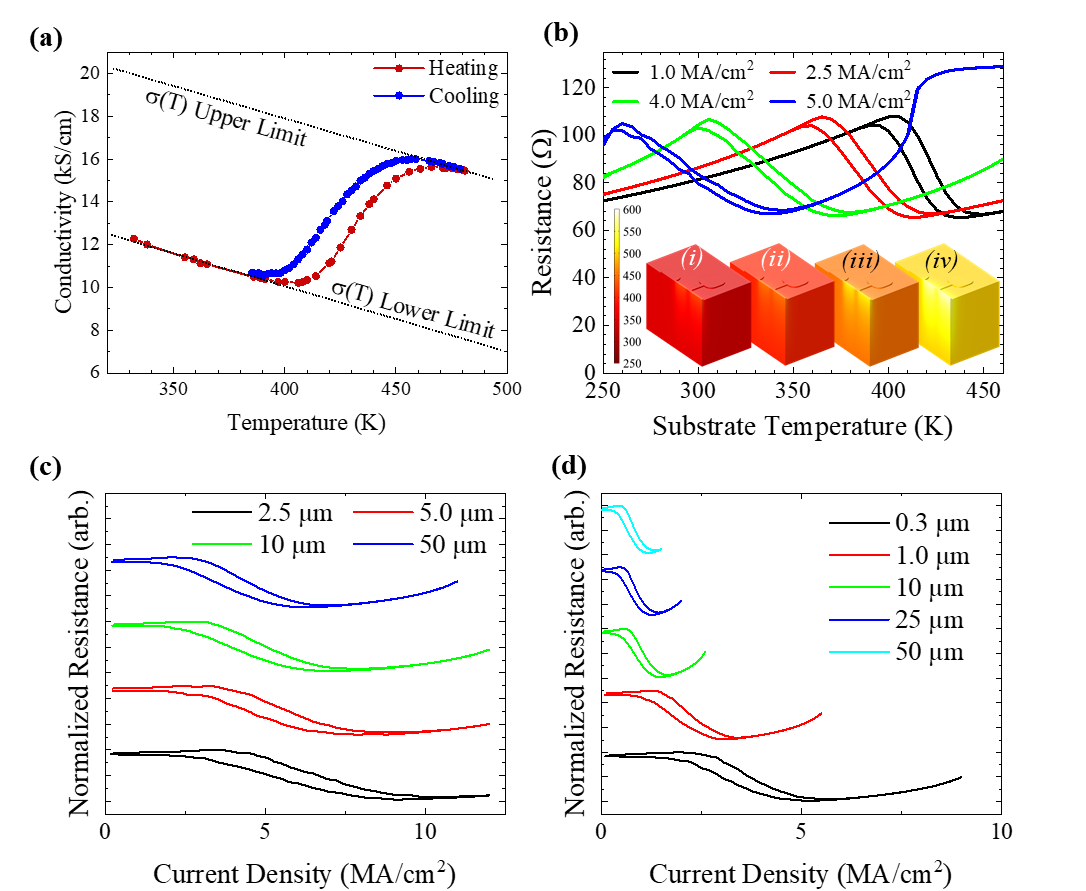


**Figure S1. (a)** Experimental conductivity measurements during heating (red), cooling (blue), and linear fits to the conductivity in AFM and FM states shown with dotted lines. **(b)** Simulated FeRh wire resistance as a function of substrate temperature for varying current densities. Inset shows MgO substrate temperature profiles while applying a current density of 4 MA/cm^2^ at fixed bottom-surface temperatures of (*i*) 350 K, (*ii*) 400 K, (*iii*) 450 K, and (*iv*) 500 K. **(c)** Simulated FeRh wire resistance as a function of applied current density for a 1-μm-wide wire with varying length. **(d)** Simulated FeRh wire resistance as a function of applied current density for a 100 μm long wire with varying width. Data shown in **(b)** and **(c)** was obtained for a substrate temperature of 400 K.

**Modeling of wire temperature profile.**

Further analysis of the model results are provided in Fig. S2, including FeRh wire temperature profile within the FeRh wire (x-axis positions ≤ |50| μm) and extended into the Au contacts on either side (x-axis positions ≥ |50| μm) at substrate temperatures of (a) 250 K and (b) 400 K. It is beyond our capabilities to measure the FeRh temperature at different points along the wire. However, this could also prove to be useful in studying the thermal mechanics of such a switching device. The modeled temperature profile within the FeRh wire is nearly constant, and referred to as a temperature plateau. Near the metal contacts (within ± 50 µm) the temperature abruptly decreases as the temperature equilibrates with the substrate temperature. This effect is consistent until exceeding a distance of ~ 25 µm from the metal/FeRh interface. Joule heating causes the plateau amplitude to increase and also causes plateau rounding near the metal/FeRh interface. During the thermal cycling simulation, one can envision these profiles as being shifted vertically toward higher temperatures. Then upon reaching the metamagnetic transition temperature, the rate at which this translation occurs begins to slow since the wire resistance and power dissipated by Joule heating (*I^2^R*) decreases. This is indeed what we observed when analyzing the first derivative of the terminal resistance with respect to temperature as shown in Fig. S2c. Generally, we see that *dR/dT* > 0 due to the positive temperature coefficient of resistance. However, as the wire transitions from AFM to FM phase, we observe an abrupt decrease in *dR/dT* such that it is less than 0. Further increasing the substrate temperature, we see *dR/dT* > 0 once the AFM-FM transition is complete. For I = 0.60 mA, the *dR/dT* transition window is relatively abrupt and the width (T = 395 to 455 K) greatly exceeds the plateau temperature of the wire. Therefore, the *dR/dT* for this low current sweep is effectively tracing the probability distribution of a domain in the AFM state to transition to the FM state. Upon increasing the current we see the *dR/dT* minima shift to lower temperatures since less substrate heating is needed to reach the transition. As shown in Fig. S2c, these various


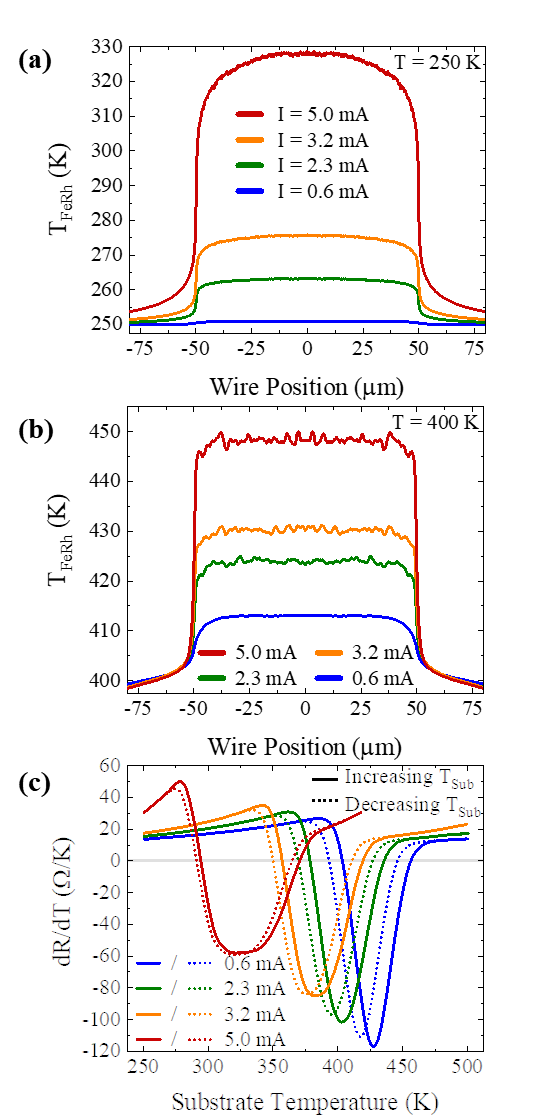


**Figure S2.** Position-dependent temperature profile for substrate temperatures of **(a)** 250 K and **(b)** 400 K. The data shown here corresponds to that of Fig. S1b for varying current amplitudes. These findings indicate significant heatsink-effects at the metal/FeRh interface. **(c)** The first derivative of resistance with respect to temperature, dR/dT, using data from Fig. S1b. Here we observe a ‘plateau’ region which widens as the current amplitude in increased.

trends correlate quite well. Broadening of *dR/dT* transition region is in part caused by the increased curvature of the temperature plateaus near the metal/FeRh interface. Away from the metamagnetic transition, the resistance should vary linearly with temperature, leading to a constant *dR/dT*. However, a slight linear increase is observed in the data. This linear increase is caused by the thermal feedback in the model where an increased substrate temperature causes an increased resistance, which causes increased Joule heating. More Joule heating increases the local wire temperature and further increases the resistance. The increased local resistance is scaled by *I^2^* and therefore increases with current density.

**Transient Modeling.**

Figure S3a shows simulation results where a 30 V voltage pulse of 240 ns pulse width and *V_b_* of 0 V is applied to the wire. Heat dissipation time constants can be extracted from the transient profile using

$$T_{rise}=T_{0}+A_{f}e^{-\frac{x}{\tau_{rf}}}+A_{s}e^{-\frac{x}{\tau_{rs}}} (9)$$

and

$$T_{decay}=T_{0}+B_{f}e^{-\frac{x}{\tau_{df}}}+B_{s}e^{-\frac{x}{\tau_{ds}}}, (10)$$

where the time constant abbreviations of *r*, *d*, *f*, and *s* represent rise, decay, fast, and slow, respectively. The time constants were found to be $\tau_{rf}=\tau_{rs}=200.7 ns$, $\tau_{df}=21.8 ns$, and $\tau_{ds}=587.2 ns$. This means that when a 30 V bias is applied with *V_b_* = 0 V, the wire temperature is elevated through Joule heating by nearly 40 K over 200 ns. Then, upon removing the 30 V bias, the heat is dissipated from the wire in 587 ns, with approximately 87.5% of the generated heat being dissipated in just 21.8 ns.

Next, we simulate state-switching capability at substrate temperatures and baseline voltages ranging from 275 – 400 K and 12 – 20 volts, respectively, by applying the pulsed waveforms shown in Fig. S3b. The findings are summarized in Fig. S3c and the transient resistance and FeRh wire temperature plots are shown in Figs. S3d – S3o. At temperature of 275 K (d, e) and 300 K (f, g) the FeRh was unable to switch into the FM state regardless of V_b_ due to the low substrate temperature. At 325 K (h, i) AFM-FM modulation begins at V_b_ = 20 V, but the low substrate temperature allows the FeRh to cool back into the AFM state when no switching bias is applied. State-switching is observed when increasing the substrate temperature to 350 K (j, k) and 375 K (l, m), when applying baseline voltages of 18 V and 14 V, respectively. At other biasing conditions the FeRh is either locked into the AFM or FM phase. At 400 K (n, o) the FeRh immediately enters the FM phase, but is unable to switch back to AFM as the substrate temperature is too high. Figure S3c shows substrate temperature versus baseline voltage with AFM, FM, and AFM-FM modulation conditions plotted. In the T_Sub_-V_b_ regions shaded blue and red, the FeRh was locked in the AFM and FM phases, respectively. However, in-between these two regions we see AFM-FM modulation, and this region of allowed transitions can be described by

$$T\left( K \right)=462.5 K-\left( 6.25 K V^{-1} \right)V_{b} (11)$$

which was acquired by fitting as shown in the plot.


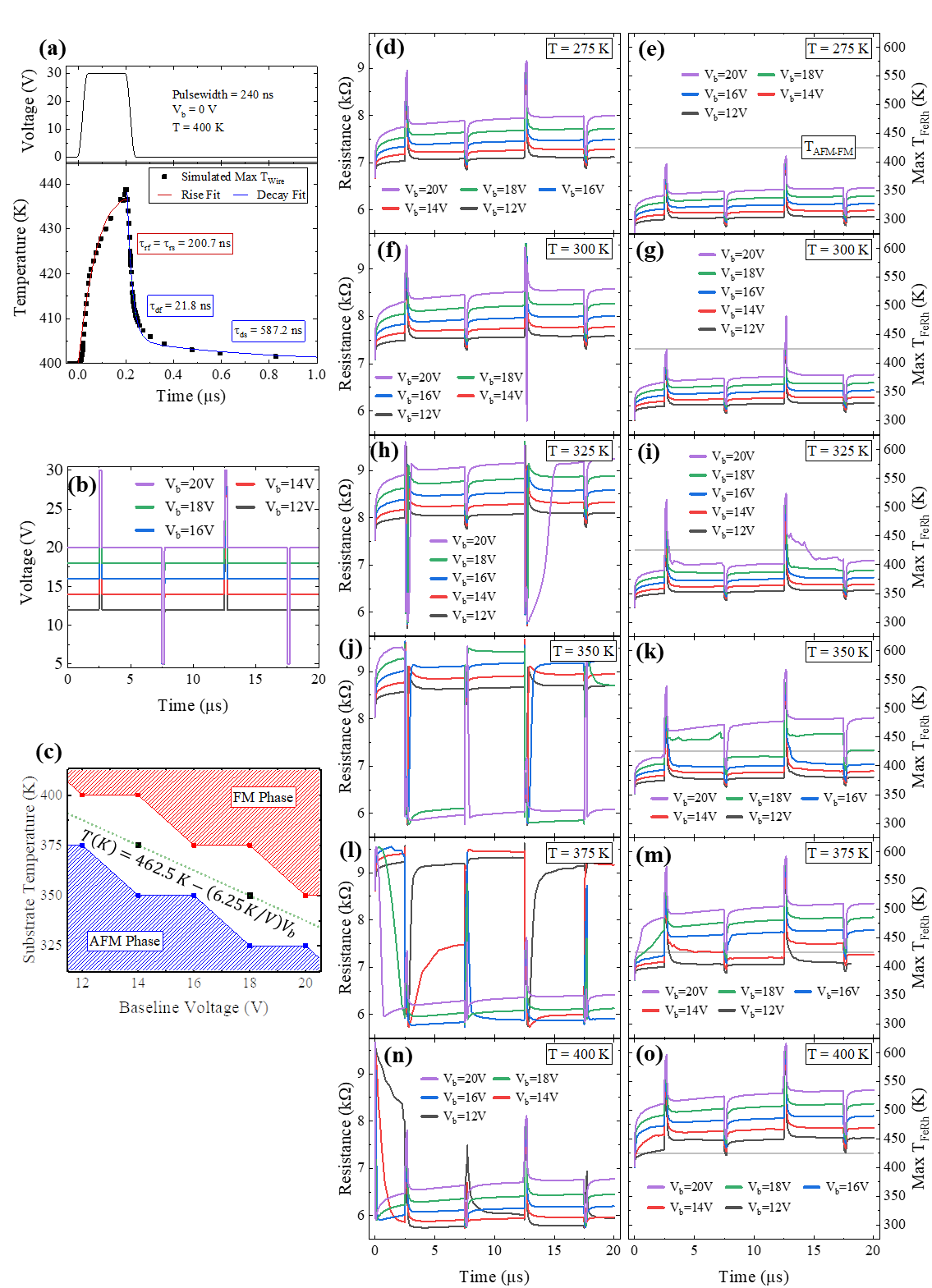


**Figure S3.** **(a)** FeRh wire heat dissipation time constant. **(b)** Simulated voltage pulse profile. **(c)** Compiled AFM-FM transition dynamics obtained from the modeled results shown in **(d)** – **(o)**. The simulated transient data includes FeRh resistance and max wire temperature.

**Switching endurance and state retention.**

Figures S4a and S4b show the device switching endurance and state retention, respectively. Endurance was evaluated using the same experimental conditions described for the measurement of Fig. 5b**.** No noticeable change in the device performance was observed when subject to more than 1x10^4^ switching cycles. State retention was also investigated by switching into the desired state then monitoring resistance as a function of time for more than one hour. In either case, the resistance reached saturation before the end of the one-hour measurement. R_OFF_ decreased by 24 Ω (0.47%) from the initial value, while R_ON_ increased by 12 Ω (0.24%) from the initial value.

*
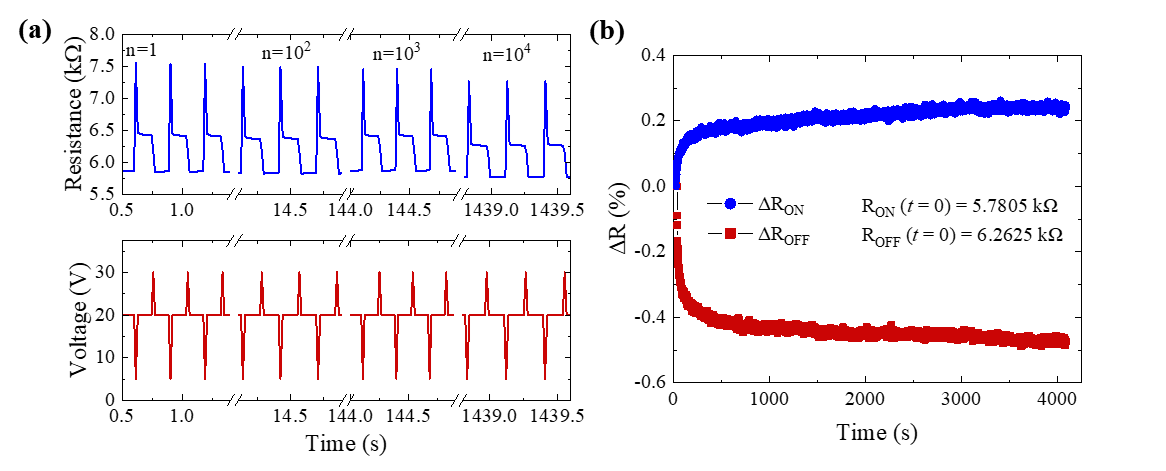
*

**Figure S4. (a)** FeRh endurance was evaluated by switching between the two device states for more than 1x10^4^ cycles. Only a minimal change in resistance was observed. **(b)** The device state retention was investigated by switching into the desired state then monitoring transient resistance for more than one hour. The resistance reached saturation before the end of the one-hour measurement. R_OFF_ decreased by 24 Ω (0.47%) from the initial value, while R_ON_ increased by 12 Ω (0.24%) from the initial value.

**REFERENCES**

[63] M. J. Jiménez, A. B. Schvval, and G. F. Cabeza, *Ab Initio Study of FeRh Alloy Properties*, Comput. Mater. Sci. **172**, 109385 (2020).

[64] D. W. Cooke, F. Hellman, C. Baldasseroni, C. Bordel, S. Moyerman, and E. E. Fullerton, *Thermodynamic Measurements of Fe-Rh Alloys*, Phys. Rev. Lett. **109**, 255901 (2012).

[65] A. B. Batdalov, A. M. Aliev, L. N. Khanov, A. P. Kamantsev, A. V. Mashirov, V. V. Koledov, and V. G. Shavrov, *Specific Heat, Electrical Resistivity, and Magnetocaloric Study of Phase Transition in Fe 48 Rh 52 Alloy*, J. Appl. Phys. **128**, 013902 (2020).

[66] A. M. Hofmeister, *Thermal Diffusivity and Thermal Conductivity of Single-Crystal MgO and Al2O3 and Related Compounds as a Function of Temperature*, Phys. Chem. Miner. **41**, 361 (2014).

[67] D. R. Lide, *CRC Handbook of Chemistry and Physics. 81st Edition* (CRC Press, 2000).
